# COVID-19, WHO guidelines, pedagogy, and respite

**DOI:** 10.1515/med-2021-0266

**Published:** 2021-03-29

**Authors:** Ranjan K. Mohapatra, Snehasish Mishra, Mohammad Azam, Kuldeep Dhama

**Affiliations:** Department of Chemistry, Government College of Engineering, Keonjhar, Odisha-758002, India; School of Biotechnology, KIIT Deemed University, Bhubaneswar, Odisha-751024, India; Department of Chemistry, College of Science, King Saud University, PO Box 2455, Riyadh 11451, Saudi Arabia; Division of Pathology, ICAR-Indian Veterinary Research Institute, Izatnagar, Bareilly-243122, Uttar Pradesh, India

Dear Editor,

The ongoing coronavirus disease 2019 (COVID-19) pandemic, allegedly caused by the so-called severe acute respiratory syndrome coronavirus 2 (SARS-CoV-2), has become a major cause of serious health concern worldwide. People are committed to staying indoors either because of a compulsion to respect prevailing local lockdown guidelines or out of fear for the need to self-protect from this seemingly never-ending pandemic. This highly contagious viral infection from China was first officially reported on 31 December 2019, and it has since affected populations in most parts of the world [1,2,3]. This emerging and evolving virus is believed to have originated from either pangolins or bats, with the latter suspected to be the major source as declared by the World Health Organization [4,5]. While COVID-19 pandemic is easy to spread but its symptoms and clinical presentations are complicated, often making it difficult to diagnose especially without any apparent epidemiological exposure and asymptomatic condition. There are reports with regard to its associated diagnosed typical [5,6] and atypical [7,8] symptoms while affecting multiple organs. The typical visible symptoms include, though not restricted to, dry cough, fever, short breath, fatigue, and dyspnea.

To date, the pandemic has adversely affected global economies significantly and consequently rewritten many socioeconomic activities on a large scale, including the stock and financial markets. Importantly, it has led to many job losses and the bankruptcy of small businesses, necessitating local government assistance in the range of billions to trillions of dollars. In addition, all facets of human life – including cultural, social, festival, and knowledge-sharing activities, to name but a few – have been affected, creating a paradigm shift in human behavioral patterns. To add to the woes, the virus has disheartened sports lovers in a big way and has even wiped out the careers of some international athletes, including those in popular sports such as tennis, football, and cricket.

However, a point of major concern here is the future generation and their education and social lives. It is our opinion that the education system has been the most affected in a way that could change the world forever. Schools, colleges, and universities in most of the affected nations worldwide are still closed and are grossly dependent on online class delivery systems, online examination/evaluation, and all other related aspects of pedagogy. The academic fraternity would universally agree more than disagree that online classes and evaluation systems are not that effective and could never replace face-to-face learning [9]. As a result, current student communities will suffer greatly, as has been made evident from their unexpected performances either way in the few bygone semesters. The percentage of marks secured by the students invariably does not really reflect their talent or potential. The deadly virus has not only taken away millions of lives and livelihoods worldwide but has also significantly added negative experiences to the teaching-learning process, thereby affecting students the most. In developing countries, the shift to online education systems has been a huge challenge owing to the lack of cyber/virtual and structural infrastructure resources and the low skill set of the majority of stakeholders for adapting to a virtual platform. This situation is direr in a huge subcontinental country like India, where the geographical vastness is an additional limiting factor. In today’s India, nearly 30% of the population is not aptly computer literate, and the majority of her educational institutions are situated in areas with limited or subpar internet services. Moreover, same situation is faced by Chinese parents as they were neither trained nor ready to embrace online learning process [10]. A high degree of technical skill and online capability is more warranted for such challenges to be addressed.

The other critical aspect in the lives of children is their behavioral pattern, as remaining behind closed doors in their homes leaves little scope for socialization. Along with issues related to their studies, children below the age of 10 years are additionally stressed as they miss out on an essential part of their lives, that is, playing in the open with their peers. Students in countries with the highest rates of infection (e.g., India, the USA, and Brazil) are the most affected. According to recent reports, schools in the state of Andhra Pradesh (India) remained opened in the months of October and November 2020 in defiance of the national lockdown guidelines, which resulted in a significant number of children getting affected through community transmission [11,12]. Moreover, 100 students at the Indian Institute of Technology (IIT), Chennai (India) were reportedly infected and tested to be SARS-CoV-2 positive in the earlier part of December 2020 after the institute had reopened [13,14]. Recently, there have been spread of a variant SARS-CoV-2 strain (UK variant B.1.1.7) in various Indian states, including in educational institutions even as they reopened for in-campus academic activities after about a year of online pedagogy. The situation at VVS Medical College in Burla, Odisha is a glaring case instance [15]. The situation is worrisome owing to the fact that in such situations the possibilities of all children obeying social distancing, regular hand-washing measures, and wearing a mask properly in their alma mater are quite low. As is currently being experienced, the opening of alma maters and playgrounds and the abilities to freely socialize and enjoy a campus life seem like a distant dream for students across all age groups.

Can there be an end to this or will the carnage continue for long? Is the education system in particular and social life in general destined to be altered forever in this era of a “new normal?” There are no answers to these questions, at least in the foreseeable future; only time will reveal the outcomes. For now, we can only stand by to await the results of whether SARS-CoV-2 or humanity wins this battle and whether the ultimate solution to saving humanity is vaccination, herd immunity through natural infection, or the complete eradication of the virus. Although there have been a silver-lining in the form of a few promising COVID-19 vaccines available now [16], these are being conditionally administered as a precondition of “emergency use,” which means their efficacy against the original SARS-CoV-2 strain is yet to be established beyond doubt. For instance, India has developed its own indigenous vaccines, Covishield and Covaxin, and vaccination program is in progress [17,18,19]. It is noteworthy that the timeline for a thorough and foolproof vaccine development is about 18 months from the day of its inception. Further, whether the developed vaccines shall be effective against the emerging mutated strain(s) of the virus is another clinical issue to address. Present situation seems to be improving as compared to the second wave of COVID-19 when the cases and deaths were increasing continuously and reached the all-time peak during January 2021 amid the ongoing pandemic. Also visible is the possibility of safe opening of schools and universities, and hopefully the ongoing vaccination program along with adopting appropriate mitigation strategies including wearing face masks, hand washing, sanitation and disinfection, social distancing, and monitoring of the SARS-CoV-2 continuously would aid in limiting the spread of COVID-19 in the coming future.
